# Pupillometry Tracks Errors in Interval Timing

**DOI:** 10.1037/bne0000533

**Published:** 2022-10

**Authors:** Shamini Warda, Jaana Simola, Devin B. Terhune

**Affiliations:** 1 Department of Humanities and Social Sciences, Indian Institute of Technology Bombay; 2 Department of Education, University of Helsinki; 3 Department of Psychology, Goldsmiths, University of London; 4 Department of Psychology, Institute of Psychiatry, Psychology and Neuroscience, King’s College London

**Keywords:** interval timing, pupillometry, error monitoring, attentional lapses

## Abstract

Recent primate studies suggest a potential link between pupil size and subjectively elapsed duration. Here, we sought to investigate the relationship between pupil size and perceived duration in human participants performing two temporal bisection tasks in the subsecond and suprasecond interval ranges. In the subsecond task, pupil diameter was greater during stimulus processing when shorter intervals were overestimated but also during and after stimulus offset when longer intervals were underestimated. By contrast, in the suprasecond task, larger pupil diameter was observed only in the late stimulus offset phase prior to response prompts when longer intervals were underestimated. This pattern of results suggests that pupil diameter relates to an error monitoring mechanism in interval timing. These results are at odds with a direct relationship between pupil size and the perception of duration but suggest that pupillometric variation might play a key role in signifying errors related to temporal judgments.

Accurate perception of time in the subsecond to suprasecond range is critical for many aspects of learning and behavior (Mauk & Ruiz, 1992; Savastano & Miller, 1998). Whereas an objective clock runs at a steady pace, our subjective time is prone to various distortions arising from factors such as attention (Brown, 1985; Mattes & Ulrich, 1998) and arousal (Penton-Voak et al., 1996). A promising biomarker to track attentional and arousal states in humans is pupil size (Mathôt et al., 2013; Murphy et al., 2011). Converging evidence suggests that changes in pupil size are an indicator of cognitive functioning reflecting a wide range of processes from visual processing to decision-making (Hess & Polt, 1960; Joshi & Gold, 2020; Mathôt, 2018; Mathôt & Van der Stigchel, 2015; Murphy, Vandekerckhove, & Nieuwenhuis, 2014).

Pupil-linked brain states, which are primarily associated with the activity in the locus coeruleus-norepinephrine (LC-NE) system (Joshi et al., 2016; Murphy et al., 2014) but may also involve cholinergic (Reimer et al., 2016), dopaminergic (de Gee et al., 2017), and serotonergic (Schmid et al., 2015) systems, can potentially influence neural activity related to the monitoring of elapsed time (Suzuki et al., 2016; Suzuki & Tanaka, 2017). In a study by Suzuki et al. (2016), a negative correlation was observed between pupil size and latency of self-timed saccades. Monkeys made a memory-guided saccade after a previously trained specified interval and analysis of pupil size with respect to saccadic latency revealed a significantly larger pupil diameter in the early saccadic latency group. Early saccadic latency suggests an expansion of perceived duration, and therefore, its correlation with pupil size was considered to be indicative of an association between pupil size and perceived duration. However, it is not yet clear whether these findings generalize to tasks that require manual responses regarding time judgments in human participants.

Using pupillometry, Toscano-Zapién et al. (2016) aimed to understand the attentional mechanisms involved in the timing of subsecond durations. Although it was not predicted in advance, pupil diameter was larger when participants incorrectly judged the duration of stimulus intervals (including “short” responses for long intervals and “long” responses for short intervals). An independent body of evidence linking pupil size and temporal information processing comes from studies of temporal expectation. Pupil size has been shown to track temporal regularities, exhibiting different preparatory activity for different delay conditions (Akdoğan et al., 2016). For example, the pupil dilates at a higher rate when targets are expected to appear after a shorter relative to a longer delay period. Additionally, pupil dilation, given its involuntary nature, has been proposed to represent a potentially valuable measure of infants’ interval timing abilities (Addyman et al., 2014).

Despite these promising links between pupil diameter and timing, there has not yet been an explicit attempt to investigate if pupillary variability relates to intra-individual variability in perceived duration in human participants. Toward this end, the present study examined whether interval timing performance covaried with pupillometry by having participants complete subsecond and suprasecond visual temporal bisection tasks while their eye movements were recorded. In turn, we sought to examine the relationship between pupil size and perceived duration of visual stimulus intervals. Following Suzuki et al (2016), we expected that pupil diameter would be larger when intervals were perceived to be longer and we explored this association at multiple phases of stimulus processing. We also evaluated the competing hypothesis that pupil size indexes error processing (Toscano-Zapién et al., 2016), rather than perceived duration per se, and tested the alternative prediction that pupil diameter would be larger when participants make incorrect interval judgments.

## Method

### Participants

A sample of 31 adults (10 females; *M*_age_ = 23.9, *SE* = 0.9) consented to take part in the study in accordance with local ethical approval from the Division of Medical Sciences, University of Oxford. All participants were right-handed, self-reported normal or corrected-to-normal vision, and had completed secondary school, with an average of 3.7 ± 0.5 years of higher education. Participants were recruited via fliers and word of mouth for an eye-tracking study on perception. These data consist of a reanalysis of a previous study that did not involve the analysis of pupillometric data (Terhune et al., 2016) and thus no formal a priori power analysis was undertaken for the present study. The original study was run to detect weak effect sizes in the range of 0.20 and above and optional stopping was not performed. Following preprocessing steps and data segregation (see below), the data from six participants were excluded from one of the two tasks (one participant had an excessively large number of blinks in both tasks, three participants had a large number of missing data points in the stimulus offset phase in suprasecond task, and two different participants in each of the tasks had perfect accuracy for at least one of the stimulus intervals), resulting in a final sample of 29 and 26 participants in the subsecond and suprasecond tasks, respectively.

### Materials

#### Temporal Bisection Tasks

Participants completed two temporal bisection tasks in subsecond and suprasecond interval ranges. All visual stimuli were presented against a purple-gray background. Intervals in the two tasks ranged from 300 to 700 ms in 63 ms increments (seven intervals) and 1,400 to 2,600 ms in 200 ms increments, respectively. Participants were initially trained to distinguish between two anchor durations (subsecond: 300 vs. 700; suprasecond: 1,400 vs. 2,600). These intervals were selected to index subsecond (∼500 ms) and suprasecond (∼2000 ms) interval timing. They were subsequently presented with variable intervals and judged whether they were closer to the trained short or long anchor intervals. Each trial consisted of a blank jittered interstimulus interval (ISI; 1,250–1,450 ms), the target interval stimulus (a centrally located light green circle), another jittered ISI (subsecond: 800–1,200 ms; suprasecond: 900–2,100 ms), and a response screen (S L; S = “short”; L = “long”) to which participants judged the duration of the interval relative to the anchor intervals using the index and middle fingers of their right hand (see Supplemental Figure 1).

### Procedure

Participants were first seated comfortably in a light-controlled, sound-attenuated room and their head was placed on a chin and forehead rest at a distance of ∼75 cm from the monitor. Eye movements and pupil data were recorded using an Eye Link 1000 Desktop Mount eye tracker (SR Research, Ontario, Canada). Data were monocularly recorded at a rate of 500 Hz from the right eye. The eye tracker was calibrated for each participant using a nine-point calibration procedure and the calibration was accepted if the average error was less than 0.5°. Participants then received onscreen and oral instructions regarding the completion of the two tasks, which were administered in counterbalanced order. They were instructed to fixate on the center of the monitor throughout the task. Each participant completed a training session of 20 trials followed by four blocks of 70 trials with randomly presented stimulus intervals. Finger-response mappings were counterbalanced across participants. The tasks were presented using Experiment Builder (v. 1.6.121; SR Research, Ontario, Canada). Stimuli subtended a visual angle of 1.73° × 1.73°. The background and stimuli were matched for luminance using a ColorCAL MkII colorimeter (Cambridge Research Systems Ltd: Rochester, United Kingdom). All participants were thanked and compensated for their time at a rate of £10/hr.

### Analyses

#### Pupillometric Preprocessing

All analyses, including statistical analyses, were performed using MATLAB (2018b, Math Works, Natick, MA). Each participant’s pupillometric data were initially segregated into two phases, prestimulus: −1,000 ms to 0 ms [stimulus onset]; poststimulus: 0 ms to response prompt [variable], and seven stimulus intervals. For each phase, a novel noise-based blink detection algorithm was used to identify start and end points for each blink (Hershman et al., 2018). Each blink period was subsequently replaced by linear interpolation of pupil diameter values prior to and after the blink period. Trials with unidentifiable blink onset/offset markers and participants missing more than 20% of data points in any of the phases were removed from the sample. There were three participants in the suprasecond task who did not have a complete set of data points in the stimulus phase and one participant in both of the tasks with a large number of missing data points in both prestimulus and stimulus phases. This resulted in the exclusion of data from one and four participant(s) in the subsecond and suprasecond tasks, respectively.

Baseline correction was performed for the stimulus phase using the median pupil diameter of the final 100 ms prior to stimulus onset. Trial-level outliers (*Mdn* ± 3*SD*) were subsequently removed using a Hampel identifier (Hampel, 1974). Data were then down-sampled by a factor of 20 (computing the mean pupil diameter for every 20 ms bin), such that each 1,000 ms window of data was represented by 50 data points per participant. The stimulus phase was subsequently segregated into three phases: stimulus onset (subsecond: 0–300 ms; suprasecond: 0–1,400 ms), stimulus preoffset (subsecond: −300 to 0 ms; suprasecond: −1,400 to 0 ms), and stimulus offset (subsecond: 0–800 ms; suprasecond: 0–900 ms).

#### Statistical Analyses

Preprocessed data for both tasks were next segregated according to responses and interval ranges collapsing across three stimulus intervals (subsecond: short: 300, 367, 433 ms, long: 567, 633, 700 ms; suprasecond: short: 1,400, 1,600, 1,800 ms, long: 2,200, 2,400, 2,600 ms). For both the prestimulus and each poststimulus (baseline)-corrected phase, and for each stimulus interval range, the mean pupil diameter corresponding to short and long responses was computed for each time bin within the respective phase. Two participants who had perfect accuracy for either short or long intervals in either task were excluded from further analyses, resulting in a final sample size of 29 and 26 for the subsecond and suprasecond tasks, respectively.

Pupil diameter in each time bin was subsequently analyzed using a series of 2 × 2 repeated-measures analyses of variance (ANOVAs) with response (short vs. long) and interval (short vs. long) as independent variables. Additionally, separate sets of ANOVAs with response (correct vs. error) and interval (short vs. long) were performed on data in the prestimulus and three stimulus phases for both tasks. For each family of tests, the statistical significance of main and interaction effects was corrected for multiple analyses using a false discovery rate (FDR) correction (Benjamini & Hochberg, 1995). Effect sizes (Hedges’s *g*) and corresponding bootstrap 95% confidence intervals (CIs; 3,000 samples) were computed for significant time windows (Hentschke & Stüttgen, 2011). This was done using the means computed from the baseline-corrected pupil diameter averaged across the significant (interaction) time window and all reported mean differences (*MDs*) reflect the mean of short responses subtracted from the mean of long response.

## Results

**Figure 1 fig1:**
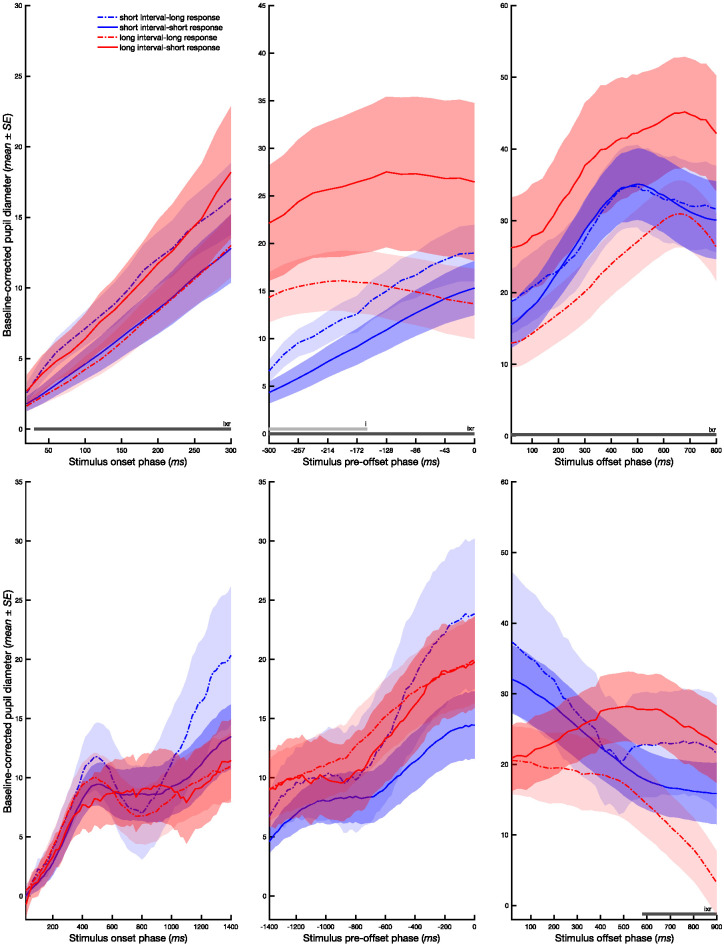
Pupil Diameter as a Function of Temporal Judgments *Note*. Baseline corrected pupil diameter during stimulus onset, stimulus preoffset, and stimulus offset phases of subsecond (top row) and suprasecond (bottom row) temporal bisection tasks as a function of stimulus interval (short vs. long) and response (short vs. long). In the three phases, 0 corresponds to stimulus onset (left), offset (middle), and offset (right), respectively. Horizontal bars denote significant (FDR corrected) interval effects (light gray) and Interval × Response interactions (dark gray). FDR = false discovery rate; SE = standard error. See the online article for the color version of this figure.

Figure 1 shows baseline-corrected average pupil diameter as a function of stimulus interval and response in each of the three phases of the two temporal bisection tasks. The analyses of the subsecond task revealed no significant main effects of response (short vs. long) at any of the time points across the three stimulus phases (Table 1). By contrast, significant main effects of interval (short < long) were found from −300 to −160 ms prior to stimulus offset, *F*s(1, 28) = 4.82–12.55, *p*s < .020, η_p_^2^s = 0.15–0.31, but not in any other phases. However, there was a clear Response × Interval interaction across all time points beginning at 20 ms from stimulus onset, *F*s(1, 28) = 5.12–8.78, *p*s < .031, η_p_^2^s = 0.16–0.24, and continuing through the stimulus preoffset and stimulus offset phases.[Fig fig1][Table tbl1]

Subsidiary analyses in the stimulus onset phase of the subsecond task revealed that there was a trend for larger pupil diameter when participants gave a long (error) response relative to a short (correct) response for the short intervals, *MD* = 2.92, *g* = 0.37, 95% CI [.06, .76]. This trend continued up to almost 40 ms prior to stimulus offset, *MD* = 3.81, *g* = 0.36, 95% CI [.11, .73], but was significant only in the stimulus preoffset phase (−280 to −40 ms). The converse trend was also observed for long intervals, greater pupil diameter for short [error] responses than long [correct] responses, but it did not achieve statistical significance following an FDR correction, −140 ms prior to stimulus preoffset: *MD* = −12.49, *g* = −0.36, 95% CI [−.67, −.07]; stimulus offset to response prompt: *MD* = −15.39, *g* = −0.38, 95% CI [−.68, −.07]. For short intervals, there was no specific trend observed in the stimulus offset phase, *MD* = 0.13, *g* = 0.00, 95% CI [−.33, .32].

In the suprasecond task, there were no significant main effects of interval or response (Table 2 and Figure 1). However, there was a significant interaction in the stimulus offset phase just prior to the response prompt (580–900 ms), *F*(1, 25) = 6.86–10.2, *p* < .01, η_p_^2^ = 0.21–0.29. The direction of the interaction is consistent with that observed in the subsecond task, but it did not survive an FDR correction. In particular, pupil diameter was greater for short than long responses for long intervals, *MD* = −16.38, *g* = −0.49, 95% CI [−.99, −.13]. For short intervals, there was no specific trend observed, *MD* = 6.25, *g* = 0.15, 95% CI [−.22, .43].[Table tbl2]

**Figure 2 fig2:**
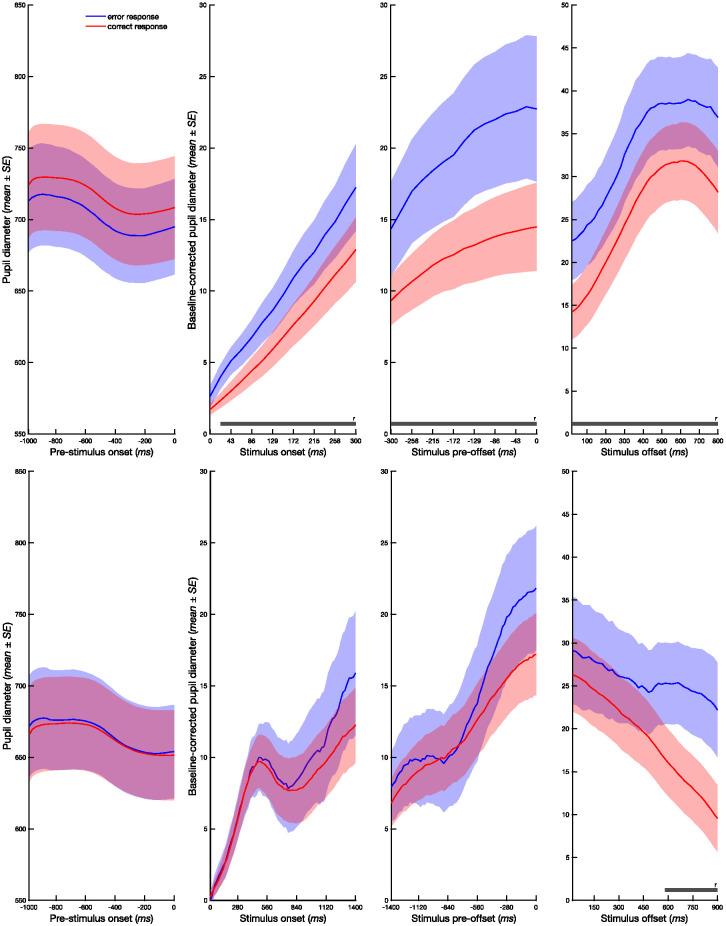
Pupil Diameter as a Function of Accuracy *Note*. Pupil diameter at baseline (and baseline corrected) during prestimulus onset, stimulus onset, stimulus preoffset, and stimulus offset phase of subsecond (top row) and suprasecond (bottom row) temporal bisection tasks as a function of response (correct vs. error). In the prestimulus and stimulus onset phases, 0 corresponds to stimulus onset and in the preoffset and offset phases, 0 corresponds to stimulus offset. Horizontal bars denote significant (FDR corrected) response effects (dark gray). FDR = false discovery rate; SE = standard error. See the online article for the color version of this figure.

A separate series of ANOVAs with response (correct vs. error) and interval (short vs.long) as independent variables revealed no significant main effects or interactions for the 1,000 ms prestimulus window in the subsecond or suprasecond task (see Supplemental Tables 1 and 2, for inferential statistics). However, as observed in the previous analysis of the subsecond task, there was a significant main effect of interval in the stimulus preoffset phase from −300 to −160 ms prior to stimulus offset, *F*s(1, 28) = 6.08–12.55, *p*s < .02, η_p_^2^s = 0.18–0.31, with larger pupil diameter for long compared to short intervals (see Figure 1). In addition, significant main effects of response were found from 20 ms following stimulus onset, *F*s(1, 28) = 5.12–8.78, *p*s < .03, η_p_^2^s = 0.15–0.24, and continuing through the stimulus preoffset, *F*s(1, 28) = 5.46–9.64, *p*s < .026, η_p_^2^s = 0.16–0.25, and stimulus offset phases, *F*s(1, 28) = 5.37–9.91, *p*s < .027, η_p_^2^s = 0.16–0.26 (Figure 2). Throughout these phases, pupil diameter was reliably larger for error than correct responses. A similar effect, albeit in a narrower time window, was observed in the suprasecond task. The ANOVAs including response (correct vs.error) and interval (short vs.long) as independent variables revealed a main effect of response in the stimulus offset phase (580–900 ms), *F*s(1, 25) = 6.86–10.21, *p*s < .01, η_p_^2^s = 0.21–0.29, with larger pupil diameter observed on error trials.[Fig fig2]

## Discussion

We sought to assess whether, and to what extent, pupillometry can be used to track the subjective perception of time (Suzuki et al., 2016; Suzuki & Tanaka, 2017). Pupil diameter did not reliably vary across stimulus intervals or temporal judgments, which is at odds with previous results pointing to pupil diameter as a potential index of perceived duration (Suzuki et al., 2016). Rather, our results suggest instead that pupil diameter tracks errors in subsecond timing, with larger pupil diameter observed on error trials. Collectively, these results suggest that pupil dilation during interval timing reflects an error monitoring mechanism but that pupil diameter does not robustly track subjective variability in the perception of duration.

The principal result of this study is that pupil diameter was greater when participants made errant temporal judgments regarding subsecond intervals. In particular, we found a reliable pattern of enlarged pupil diameter both when shorter intervals were overestimated (judged to be “long”) and when longer intervals were underestimated (judged to be “short”). This pattern was present both during stimulus presentation (stimulus onset: 20–300 ms; stimulus preoffset: −300 to 0) as well as after stimulus presentation (stimulus offset: 0–800). However, the temporal locus of these effects varied as a function of stimulus interval: Whereas pupil dilation was observed for short interval errors during stimulus presentation it was present during the stimulus preoffset and offset phases for long-interval errors. By contrast, corresponding effects were not reliably observed in a suprasecond temporal bisection task. For example, the corresponding interaction of stimulus interval and perceptual response was replicated only in the late stimulus offset phase just prior to the response prompt (580–900 ms) and was driven by differential responses for long-stimulus intervals. Taken together, these results suggest that pupil diameter during interval timing reliably indexes subsecond error monitoring mechanisms.

These results partially align with those of Toscano-Zapién et al. (2016), which suggested that pupil dilation predicts incorrect interval judgments. In their study, pupil diameter was found to be larger when participants’ gave long responses for short intervals and short responses for long intervals. More broadly, prior research suggests that pupil diameter is a reliable marker of performance prediction errors (Braem et al., 2015) and is sensitive to different types of errors according to their behavioral relevance (Maier et al., 2019). In particular, pupil dilation is observed following difficult correct trials relative to easy correct trials whereas pupil contraction is greater following difficult incorrect trials than easy incorrect trials (Braem et al., 2015). Moreover, error-related pupil dilation has been shown to be larger for perceived than unperceived errors (Wessel et al., 2011). Applied to the present data, and considering the results of Toscano-Zapién et al. (2016), a pupil dilation error monitoring mechanism might have been expected for both short- and long-stimulus intervals. However, as noted above, pupillary tracking of errors in these interval ranges varied across the phases of stimulus presentation. This potentially suggests that pupillary changes in response to timing errors are not being robustly tracked for shorter subsecond intervals, potentially because these errors are less accessible to awareness. In contrast, it is plausible that pupil dilation was more easily apparent for long-subsecond intervals because of the greater ease of detecting errors on such trials.

One another plausible explanation for the difference in pupil diameter observed during the stimulus offset phase with long-subsecond intervals is that the difference may have emerged as a consequence of lapses in attention. Previous research demonstrated that relative to self-reported on-task states, mind-wandering states were associated with temporal underestimation and increased error rates (Terhune et al., 2017). Mind-wandering is often characterized by enlarged pupil diameter (Pelagatti et al., 2018; Smallwood et al., 2011) and thus the present results potentially reflect in part errors attributable to mind-wandering or attentional lapses, such as through perceptual decoupling during mind-wandering states. Insofar as the present effect was most pronounced for longer subsecond intervals, this interpretation is arguably further bolstered by the previous finding that increased timing error rates during mind-wandering were mostly specific to long-subsecond intervals (Terhune et al., 2017). It is possible that timing errors due to attentional lapses are more likely when the response is to be given after a certain amount of time has elapsed. Further research using concurrent eye-tracking and mind-wandering state probes during interval timing is required to more rigorously evaluate this possibility.

The pattern of pupil diameter in relation to erroneous interval judgments differed across subsecond and suprasecond intervals. Although the pupil diameter was larger for incorrect interval judgments in the subsecond task, this was present in suprasecond tasks only for the poststimulus decisional phase prior to response prompts. Similar findings have been reported previously in the perceived duration of gaze shift (Binetti et al., 2017); specifically, pupil dilation associated with temporal judgments in gaze shift significantly differed only for subsecond intervals. By contrast, with suprasecond intervals, recent research suggests that there is no robust association between pupil size and temporal judgment errors (Suárez-Pinilla et al., 2019). Taken together, our results are consistent with a wealth of evidence for a functional dissociation in the mechanisms subserving subsecond and suprasecond timing (Hayashi et al., 2014; Lewis & Miall, 2003; Rammsayer & Ulrich, 2011). This work suggests that sensory-based automatic processing underlies the former whereas the latter is supported to a greater extent by executive cognitive processes. In humans, pupillary fluctuations in response to timing errors are more apparent in the subsecond range of intervals which predominantly involves lower level sensory processes.

Although the present results appear to be at odds with previous findings suggesting that pupil diameter covaries with perceived duration (Suzuki et al., 2016), these dissimilar results can potentially be reconciled. In the latter study, monkeys performed an oculomotor version of the temporal reproduction task and saccadic latency after a self-timed interval was used as an index of reproduced duration. Monkeys were trained to generate a saccade within 1,000–1,700 ms of the cue onset, of which latencies below 1,000 ms constituted nearly 30% of trials. Saccadic latencies were segregated into early, middle, and late latencies, and pupil diameter was compared between early and late latencies. Given that nearly one-third of trials included latencies shorter than the minimum trained interval (1,000 ms) and rarely any latencies were longer than the maximum interval range (1,700 ms), a large fraction of saccades clustered under the short-latency group included latencies shorter than 1,000 ms. Those saccades were not impulsive but rather were produced earlier in time, and therefore constitute errors. Should this be the case, the purported effect of increased pupil diameter in shorter latencies, suggesting subjective temporal dilation, might be indicative of an error monitoring mechanism similar to that observed here.

Our findings on interval-specific, error-related pupillary variations indirectly support the possible involvement of norepinephrine modulators in signifying errors related to the perception of duration. As such, it is reasonable to assume that the LC-NE system might play a key role in signifying errors related to temporal judgments. Given that NE is a key neuromodulator in probing attentional lapses (Smith & Nutt, 1996) and error monitoring mechanisms (Murphy et al., 2011), NE might likely mediate their influence on timing errors. The LC-NE system could possibly have different duration-sensitive channels which may have contributed to the interval-specific difference in pupil size. However, given that there was no control task to test whether the purported effect reflects a temporal error monitoring mechanism or a generic error monitoring mechanism (see, e.g., Coull et al., 2004), more research is required to determine whether the effect observed here is specific to temporal judgment errors or reflective of a broader error monitoring mechanism. Additionally, we did not analyze for any confounding or interacting effect of saccades; nevertheless, it seems unlikely that trial-by-trial variability in saccades would be a potential confound in the error-related pupil dilation observed here. Finally, the present data are unable to determine whether the observed effect is specific to the LC-NE system given that pupillometric variation has also been linked to the cholinergic, dopaminergic, and serotonergic systems (Reimer et al., 2016; Schmid et al., 2015). Further research coupling eye-tracking and pharmacological interventions targeting these systems (e.g., Coull et al., 2012) is required to further distinguish the roles of these systems in the effects observed here.

In summary, our results suggest that pupil diameter does not track the subjective perception of duration, but instead tracks errors in interval timing. This effect appears to be more specific to the processing of subsecond intervals and was most pronounced for longer subsecond intervals. We propose that LC-NE signaling underlies this pattern of pupillary dilation by its modulatory influence on mechanisms such as error monitoring and attentional lapses.

## Supplementary Material

10.1037/bne0000533.supp

## Figures and Tables

**Table 1 tbl1:** Summary Inferential Statistics for Analyses of Variance on Pupil Diameter Ranges for Different Phases of the Subsecond Temporal Bisection Task (N = 29)

Stimulus phase	*F*(1, 28)	*p*	η_p_^2^
Interval			
Prestimulus (−1,000 to 0 ms)	0.27–1.34	.25–.60	.01–.04
Stimulus onset (0 to 300 ms)	0.00–0.43	.51–.98	.00–.01
Stimulus preoffset (−300 to 0 ms)	0.55–12.55	.001–.46*	.01–.31
Stimulus offset (0 to 800 ms)	0.00–1.17	.28–.97	.00–.04
Response (short vs. long)			
Prestimulus (−1,000 to 0 ms)	0.99–2.24	.14–.32	.03–.07
Stimulus onset (0 to 300 ms)	0.00–0.14	.71–.99	.00–.004
Stimulus preoffset (−300 to 0 ms)	0.98–1.65	.21–.33	.03–.05
Stimulus offset (0 to 800 ms)	1.13–2.79	.10–.29	.04–.09
Interval × Response			
Prestimulus (−1,000 to 0 ms)	1.44–4.78	.037–.24	.04–.14
Stimulus onset (0 to 300 ms)	1.75–8.78	.006–.19*	.06–.24
Stimulus preoffset (−300 to 0 ms)	5.46–9.64	.004–.026*	.16–.26
Stimulus offset (0 to 800 ms)	5.38–9.92	.003–.027*	.16–.26
*Note*. Values reported included ranges of *F*s, *p*s, and η_p_^2^s in the respective stimulus phases.			
* At least 53.3% or more of *p* values are significant after a false discovery rate (FDR) correction, *p* < .05.			

**Table 2 tbl2:** Summary Inferential Statistics for Analyses of Variance on Pupil Diameter Ranges for Different Phases of the Suprasecond Temporal Bisection Task (N = 26)

Stimulus phase	*F*(1, 25)	*p*	η_p_^2^
Interval			
Prestimulus (−1,000 to 0 ms)	0.82–2.05	.16–.37	.03–.07
Stimulus onset (0 to 1,400 ms)	0.00–2.41	.13–.99	.00–.09
Stimulus preoffset (−1,400 to 0 ms)	0.00–1.19	.28–.95	.00–.04
Stimulus offset (0 to 900 ms)	0.00–5.01	.034–.99	.00–.16
Response (short vs. long)			
Prestimulus (−1,000 to 0 ms)	0.81–2.26	.14–.37	.03–.08
Stimulus onset (0 to 1,400 ms)	0.00–1.78	.19–.97	.00–.06
Stimulus preoffset (−1,400 to 0 ms)	0.25–1.00	.32–.62	.00–.03
Stimulus offset (0 to 900 ms)	0.00–1.25	.27–.99	.00–.05
Interval × Response			
Prestimulus (−1,000 to 0 ms)	0.01–0.59	.44–.91	.00–.02
Stimulus onset (0 to 1,400 ms)	0.00–0.52	.47–.99	.00–.02
Stimulus preoffset (−1,400 to 0 ms)	0.00–0.83	.37–.99	.00–.03
Stimulus offset (0 to 900 ms)	0.21–10.21	.003–.64*	.01–.29
*Note*. Values reported included ranges of *F*s, *p*s, and η_p_^2^s in the respective stimulus phases.			
* At least 33.3% or more of *p* values are significant after a false discovery rate (FDR) correction, *p* < .05.
