# Supplementary material for: Pupillometry Tracks Errors in Interval Timing
Source: Behav Neurosci. 2022 Oct;136(5):495–502. doi: 10.1037/bne0000533 (PMC9552500; doi:10.1037/bne0000533)
Supplement: Supplementary file 1 [file bne0000533_pupil_timing_R1_020622_SUPPL.docx]

**SUPPLEMENTARY INFORMATION**

**Pupillometry tracks errors in interval timing**

Shamini Warda^1^, Jaana Simola^2^, & Devin B. Terhune^3^

^1^Department of Humanities and Social Sciences, Indian Institute of Technology Bombay, Mumbai, India

^2^Department of Education, University of Helsinki, Finland

^3^Department of Psychology, Goldsmiths, University of London, London, UK

*Correspondence:*

Dr. Devin B. Terhune

Department of Psychology

Goldsmiths, University of London

New Cross, London, UK

d.terhune@gold.ac.uk

^
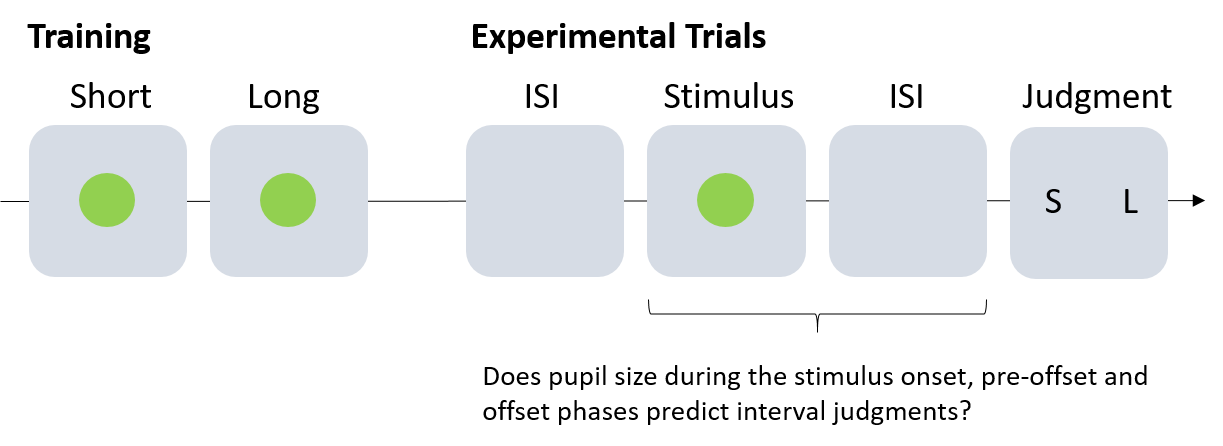
^

**Supplementary Figure 1**. *Schematic diagram of the temporal bisection tasks and the coding of pre-stimulus and three stimulus phases. The short and long intervals were segregated by collapsing across three stimulus intervals (subsecond: short: 300, 367, 433ms, long: 567, 633, 700ms; suprasecond: short: 1400, 1600, 1800ms, long: 2200, 2400, 2600ms).* *The three stimulus phases corresponds to stimulus onset (subsecond: 0-300ms; suprasecond: 0-1400ms), stimulus pre-offset (subsecond: -300-0ms; suprasecond: -1400-0ms), and stimulus offset (subsecond: 0-800ms; suprasecond: 0-900ms). For each phase, the data were segregated based on short and long responses.*

| **Supplementary Table 1**  *Summary inferential statistics for analyses of variance on pupil diameter ranges for different phases of the subsecond temporal bisection task* (*N*=29) | | | |
| --- | --- | --- | --- |
| Stimulus phase | *F*(1,28) | *p* | *η_p_^2^* |
| Interval |  |  |  |
| Prestimulus (-1000-0ms) | 0.27-1.34 | .25-.60 | .01-.04 |
| Stimulus onset (0-300ms) | 0.00-0.43 | .51-.98 | .00-.01 |
| Stimulus pre-offset (-300-0ms) | 0.55-12.55 | .001-.46* | .01-.31 |
| Stimulus offset (0-800ms) | 0.00-1.17 | .28-.97 | .00-.04 |
| Response (correct vs. incorrect) |  |  |  |
| Prestimulus (-1000-0ms) | 1.44-4.78 | .037-.24 | .04-.14 |
| Stimulus onset (0-300ms) | 1.75-8.78 | .006-.19* | .05-.23 |
| Stimulus pre-offset (-300-0ms) | 5.46-9.64 | .004-.026* | .16-.25 |
| Stimulus offset (0-800ms) | 5.37-9.91 | .003-.027* | .16-.26 |
| Interval x Response |  |  |  |
| Prestimulus (-1000-0ms) | 0.99-2.24 | .14-.32 | .03-.07 |
| Stimulus onset (0-300ms) | 0.00-0.14 | .71-.99 | .00-.004 |
| Stimulus pre-offset (-300-0ms) | 0.98-1.65 | .21-.33 | .03-.05 |
| Stimulus offset (0-800ms) | 1.13-2.79 | .10-.29 | .03-.09 |

*Notes*. Values reported included ranges of *F*s, *p*s, and *η_p_^2^*s in the respective stimulus phases.

* At least 53.3% or more of *p*-values are significant after a false discovery rate (FDR) correction, *p*<.05.

| **Supplementary Table 2**  *Summary inferential statistics for analyses of variance on pupil diameter ranges for different phases of the suprasecond temporal bisection task* (*N*=26) | | | |
| --- | --- | --- | --- |
| Stimulus Phase | *F* (1,25) | *p* | *η_p_^2^* |
| Interval |  |  |  |
| Prestimulus (-1000-0ms) | 0.82-2.05 | .16-.37 | .03-.07 |
| Stimulus onset (0-1400ms) | 0.00-2.41 | .13-.99 | .00-.08 |
| Stimulus pre-offset (-1400-0ms) | 0.00-1.19 | .28-.95 | .00-.04 |
| Stimulus offset (0-900ms) | 0.00-5.01 | .034-.99 | .00-.16 |
| Response (correct vs. incorrect) |  |  |  |
| Prestimulus (-1000-0ms) | 0.01-0.59 | .44-.91 | .00-.02 |
| Stimulus onset (0-1400ms) | 0.00-0.52 | .47-.99 | .00-.02 |
| Stimulus pre-offset (-1400-0ms) | 0.00-0.83 | .37-.99 | .00-.03 |
| Stimulus offset (0-900ms) | 0.21-10.21 | .003-.64* | .01-.29 |
| Interval x Response |  |  |  |
| Prestimulus (-1000-0ms) | 0.81-2.26 | .14-.37 | .03-.08 |
| Stimulus onset (0-1400ms) | 0.00-1.78 | .19-.97 | .00-.06 |
| Stimulus pre-offset (-1400-0ms) | 0.25-1.00 | .32-.62 | .00-.03 |
| Stimulus offset (0-900ms) | 0.00-1.25 | .27-.99 | .00-.04 |

*Notes*. Values reported included ranges of *F*s, *p*s, and *η_p_^2^*s in the respective stimulus phases.

* At least 35% or more of *p*-values are significant after a false discovery rate (FDR) correction, *p*<.05.
